# 3D Printing of Artificial Blood Vessel: Study on Multi-Parameter Optimization Design for Vascular Molding Effect in Alginate and Gelatin

**DOI:** 10.3390/mi8080237

**Published:** 2017-07-31

**Authors:** Huanbao Liu, Huixing Zhou, Haiming Lan, Tianyu Liu, Xiaolong Liu, Hejie Yu

**Affiliations:** 1 College of Engineering, China Agricultural University, Beijing 100083, China; leaderliu@cau.edu.cn (H.L.); bs20153070217@cau.edu.cn (H.L.); perc_lty@cau.edu.cn (T.L.); perc_lxl@cau.edu.cn (X.L.); yuhejie@cau.edu.cn (H.Y.); 2 School of Mechanical-Electronic Vehicular Engineering, Beijing University of Civil Engineering and Architecture, Beijing 100044, China

**Keywords:** cardiovascular disease, 3D printing, alginate–gelatin, optimized parameters

## Abstract

3D printing has emerged as one of the modern tissue engineering techniques that could potentially form scaffolds (with or without cells), which is useful in treating cardiovascular diseases. This technology has attracted extensive attention due to its possibility of curing disease in tissue engineering and organ regeneration. In this paper, we have developed a novel rotary forming device, prepared an alginate–gelatin solution for the fabrication of vessel-like structures, and further proposed a theoretical model to analyze the parameters of motion synchronization. Using this rotary forming device, we firstly establish a theoretical model to analyze the thickness under the different nozzle extrusion speeds, nozzle speeds, and servo motor speeds. Secondly, the experiments with alginate–gelatin solution are carried out to construct the vessel-like structures under all sorts of conditions. The experiment results show that the thickness cannot be adequately predicted by the theoretical model and the thickness can be controlled by changing the parameters. Finally, the optimized parameters of thickness have been adjusted to estimate the real thickness in 3D printing.

## 1. Introduction

Cardiovascular disease is a common disease that seriously affects the health of human beings, especially those over 50 years old. Many scientists [1,2,3,4,5,6] have adopted a lot of measures to treat cardiovascular disease, but more than 50% of survivors cannot completely take care of themselves. The number of deaths from cardiovascular diseases is up to 15 million people each year globally, which top killer of human beings. Therefore, it is significant to develop an effective method to fabricate vessel-like structures to treat cardiovascular disease.

3D printing is a novel technology to be applied to regenerative medicine that has attracted extensive attention due to its potential to cure disease through tissue engineering and organ regeneration [7,8,9,10,11,12]. In the past few years, many functional tissues and organs were manufactured by 3D printing systems such as inkjet-based printing [13,14,15], micro-extrusion printing [16], and laser-assisted printing [17,18,19], which have different forming parameters in different 3D printing system. One important challenge is the material viscosity characteristic which can determine the types of 3D printers. The inkjet-based printing has viscosity a limit ranging from 3.5 to 12 mPa·s and the viscosity limit of laser-assisted printing ranges from 1 to 300 mPa·s, but the viscosity limit of extrusion-based printing ranges from 30 to >6 × 10^7^ mPa·s. The different working mechanisms and parameters of nozzles results in different viscosity limits.

Many research teams have adopted the above 3D printing strategies to fabricate vessel-like structures with deposition modeling methods [20,21,22]. Cui, X. et al. [23] have used thermal inkjet printing to fabricate the human microvasculature. Then Hoch, E. et al. [24] have shown that inkjet-based 3D bioprinting can be expected to be of great use for vessel-like structures. Gaebel, R. et al. [25] have adopted laser printing to distribute the endothelial cells to fabricate blood vessels. Kucukgul, Can et al. [26] have adopted a deposition modeling method to construct vessel-like structures by extrusion-based 3D bioprinting. In those methods, the vessel-like structures have poor modeling effect and material accumulation phenomena. However, there is little research into adopting the rotary forming method to fabricate vessel-like structures. Gao, Q. et al. [27] have adopted the rotary printing method to fabricate vessel-like structures; it can proven that this method can improve the cell survival rate. Meanwhile, Liu, H. et al. [28,29] have adopted the rotary printing method to obtain vessel-like structures and analyzed the synchronization among nozzle extrusion, nozzle speed, and rotating speed based on an extrusion-based 3D bioprinter, which could improve the modeling effect in this forming method.

In this paper, we present our 3D printing device for fabricating vessel-like structures, and further optimize the thickness parameters of nozzle extrusion speed nozzle speed and rotating speed. The nozzle moving speed parameter δ and the pressure parameter ∇ have been adjusted to estimate the real thickness in the experiment.

## 2. System and Process

**Figure 1:**
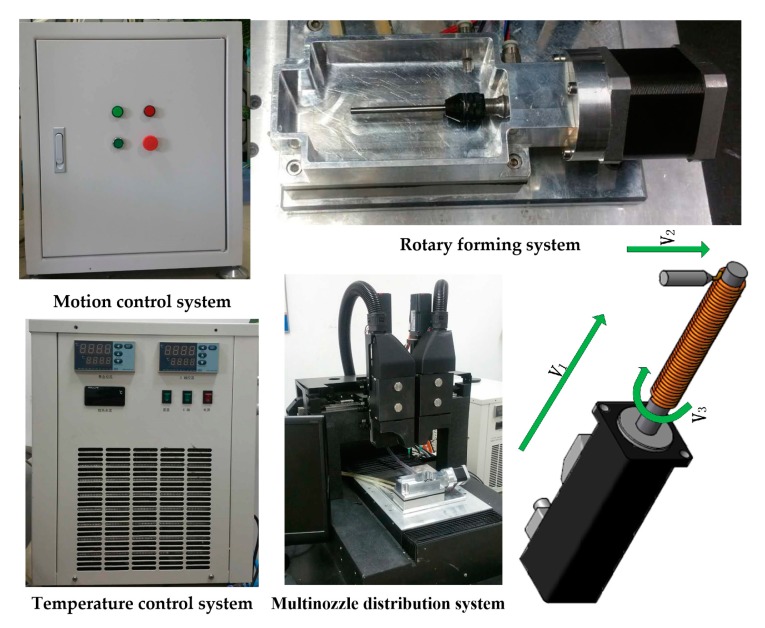
The extrusion-based 3D printing system with rotary printing device.

Figure 1 shows the extrusion-based 3D printing system, which is mainly composed of a motion control system, multinozzle distribution system, rotary forming system, and temperature control system. The multinozzle 3D printing system is a solid free-form fabrication system that performs extrusion-based processes. Two nozzles with different biomaterials can work effectively in this 3D printing system which can be controlled by computer. The specification of the 3D printing system is shown in Table 1.

In this 3D printing system, 3D physical models (STL) were divided into different regions, which were sliced to generate the G-code in the computer, and then each region was filled with different materials. The STL files can be obtained by CAD/CAM software or 3D scanners.

It is difficult to extrude biomaterials with a solid state or high viscosity. In order to reconstruct the digital model, the materials should be transformed into a liquid state or to a proper viscosity to ensure extrusion of biomaterials in the process of 3D printing. To that end, the biomaterials firstly have the characteristic of thermosensitivity, which can be liquefied in suitable temperatures (35–40 °C). Secondly, the temperature control system can control the material temperature to achieve the conversion between solid and liquid states. In addition, there is no heating damage for bioactivity in this temperature control system.

## 3. Theory

**Figure 2:**
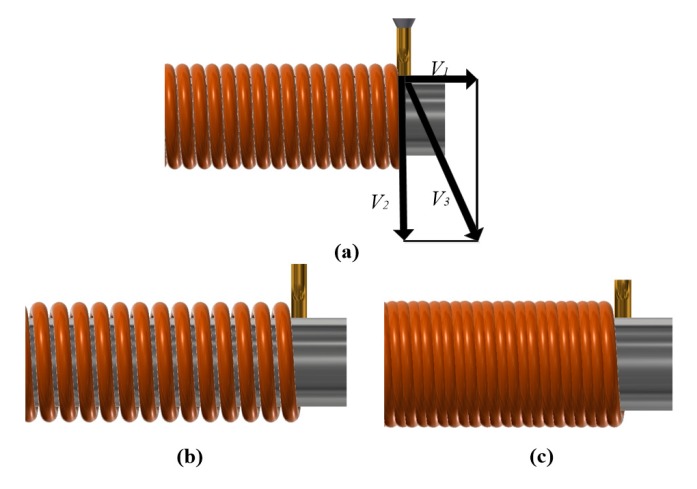
The schematic diagram of rotary printing device under the different nozzle speed: (**a**) ideal state; (**b**) nozzle moving speed greater than *V*_1_; (**c**) nozzle moving speed less than *V*_1_.

Figure 2 shows the schematic diagram of rotary printing device under the different nozzle speed. The biomaterials can be squeezed by pressure control system, the rotating rod is controlled by the motor, and the nozzle speed is controlled by linear motor. In the Figure 2a, *V*_1_, *V*_2_, and *V*_3_ denote the nozzle speed, the extrusion speed, and the motor speed respectively. When the nozzle speed is greater than *V*_1_, the biomaterials will demonstrate the dispersion phenomenon, as shown in Figure 2b. When the nozzle speed is less than *V*_1_, the biomaterials will demonstrate accumulation, as shown in Figure 2c.

In order to realize the reconstruction of vessel-like structures and avoid the phenomenon of material accumulation and material dispersion, the theoretical model can be obtained by this rotary printing device, which is given that [28]

(1) $$\vec{V_{3}}=\vec{V_{1}}+\vec{V_{2}}$$

Because equal time is consumed in the building process of vessel-like structures, the relationship among nozzle speed, extrusion speed, and motor speed is calculated as

(2) $$\frac{S_{1}}{V_{1}}=\frac{S_{2}}{V_{2}}=\frac{S_{3}}{V_{3}}$$

where $S_{1}$, $S_{2}$, and $S_{3}$ refer to linear motor displacement, rotating rod perimeter, and material extrusion displacement.

The thickness can be derived by Equations (1) and (2)

(3) $$\begin{array}{c} H=\alpha \sqrt{{\left(V_{3}L/2V_{1}\right)}^{2}-L^{2}}-r\\ H=\alpha \sqrt{{\left(V_{3}C/2V_{2}\right)}^{2}-L^{2}}-r \end{array}\}$$

where H is the thickness, L is the displacement of nozzle, α is an invariant constant, and *r* is the rotating rod radius.

**Figure 3:**
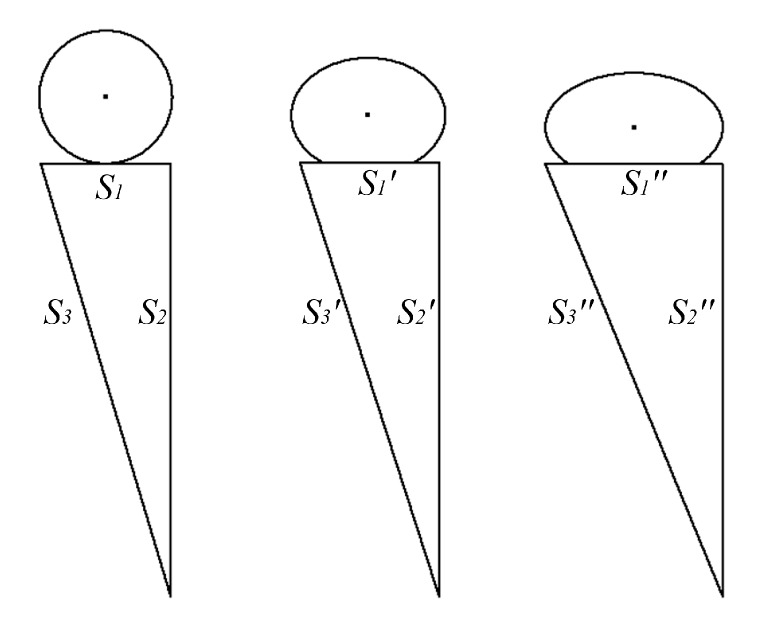
The relationship between the displacement and thickness under the different nozzle moving speed due to surface wettability.

The extrusion forming principle under the different nozzle moving speeds is shown in Figure 3. The thickness can be changed under the different control parameters. When the diameter of rotating rod and motor rotation speed remain unchanged, the displacement relation under the different nozzle moving speed can be calculated as

(4) $$\begin{array}{c} {S_{1}}^{\prime \prime }>{S_{1}}^{\prime }>S_{1}\\ {S_{2}}^{\prime \prime }={S_{2}}^{\prime }=S_{2}\\ {S_{3}}^{\prime \prime }>{S_{3}}^{\prime }>S_{3} \end{array}\}$$

The goal is to predict displacement changes which have different forming effects under different nozzle moving speeds. In order to obtain the real thickness, the new thickness coefficient can be adjusted to measure the thickness range.

## 4. Experiment Procedure

### 4.1. Materials

In order to optimize the above mentioned theoretical model, we designed the experiment to fabricate the vessel-like structures. The rotating rods with different diameters of 3, 4.26, and 6.9 mm were used in this experiment. We selected alginate and gelatin as experiment materials. In the experiment, alginate and gelatin were premixed, extruded, and post crosslinked after depositing on the rotating rod in the 3D printing system. The alginate and gelatin solution was cross-linked with calcium chloride (5% *w*/*w*). The parameters of experiment materials are shown in Table 2. All of the above materials were purchased from Xilong Scientific Co., Ltd. (Guangdong, China).

### 4.2. Experimental Procedure

The novel 3D printing system has been designed for the preliminary experiment. In this 3D printing system, the temperature control system can control the temperature of biomaterials in a range from 2 °C to 45 °C. Then, the motion control system can control the nozzle motion according to G-code document. Next, the biomaterials can be extruded by pressure control system ranged from 0.1 to 1 MPa. Finally, the biomaterials are extruded and distributed by the above-mentioned motion control system and pressure control system. Based on this, vessel-like structures can be constructed in this 3D printing system.

In order to verify the theoretical model, we used a rotating rod with different diameters to analyze the motion synchronization and the related parameters of molding effect. In this experiment, the rotating rod was set to 4.25 r/s and the moving speed of nozzle ranged from 0.8 to 8.3 mm/s. The pressure was set to 0.168–0.421 MPa, which could achieve biomaterial extrusion with different extrusion speeds.

## 5. Results and Discussion

### 5.1. Material Viscosity

**Figure 4:**
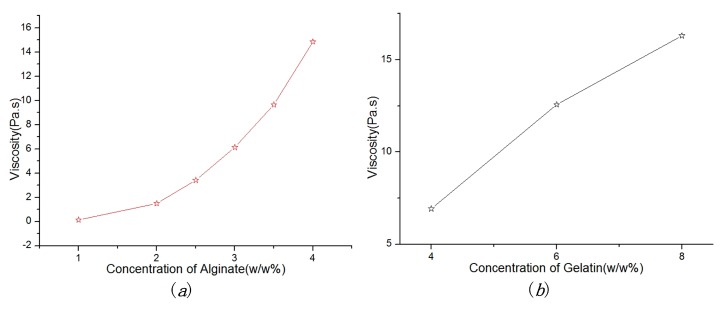
Dependence of material concentration on viscosity: (**a**) the relationship between viscosity and concentration of alginate; (**b**) the relationship between viscosity and concentration of gelatin under the concentration of alginate (3% *w*/*w*).

Viscosity is an important factor which plays a major role in fabricating the vessel-like structures. Figure 4 shows the relationship between viscosity and material concentration. In Figure 4a, the viscosity of alginate solution increases with the increase of alginate concentration. In Figure 4b, we can see that the 3% alginate with different concentrations of gelatin (4%, 6% and 8% *w*/*w*) have different viscosity characteristics. The viscosity value increased gradually along with the increase of gelatin concentration. Therefore, the alginate solution with gelatin can improve the material characteristics such as viscosity, mechanical properties, and printable.

**Figure 5:**
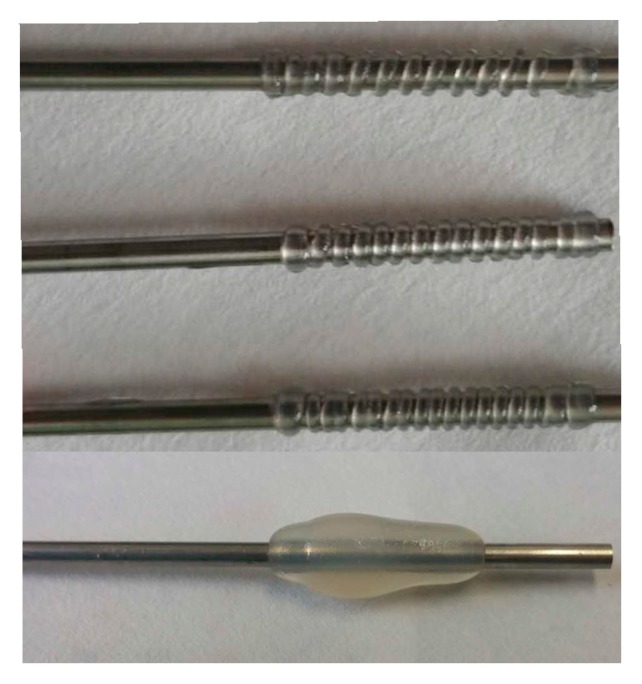
The problems of material dispersion and material accumulation.

In this experiment, the extrusion-based nozzle has been developed to fabricate the vessel-like structures. Compared with the deposition modeling, this method does not build the scaffold to sustain the vessel-like structures, which can reduce time consumption in the build process of vessel-like structure. In order to improve the forming effect, the adaptive parameters should be set to avoid the problems of material dispersion and material accumulation, as shown in Figure 5. Those problems will reduce the forming accuracy and mechanical properties.

**Figure 6:**
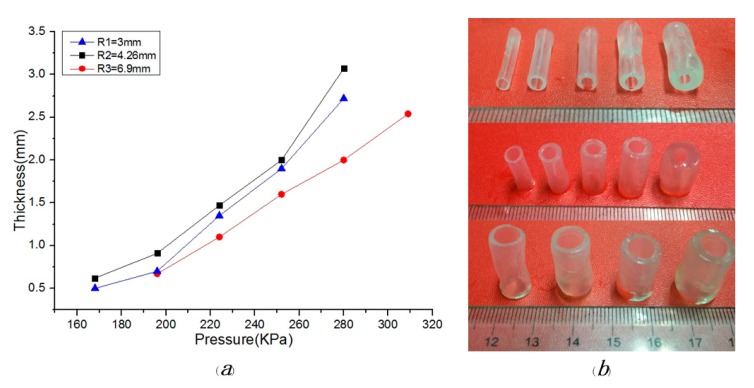
The molding relationship and effect: (**a**) dependence of the extrusion pressure on thickness under different diameters; (**b**) the relationship between molding effect and diameter.

Dependence of the extrusion pressure on molding effect: In this experiment, we have adopted a solution with 3% alginate and 8% gelatin for initial research. When the rotating speed was 4.25 r/s and nozzle moving speed was 1.62 mm/s, the vessel-like structures exhibited different molding effects under different pressures, as shown in Figure 6. The different external diameters or thicknesses can be achieved under the different extrusion pressures. Figure 6a shows the relationship between thickness and extrusion pressure. We can see that the thickness is on the increase with the increase of pressure. The molding effects are shown in Figure 6b. When the pressure exceeds the 0.31 Mpa, the molding effect appears as material accumulation, which leads to a poor forming effect. Conversely, when the pressure is below the 0.16 MPa, the vessel-like structures will not be fabricated by this 3D printing system.

**Figure 7:**
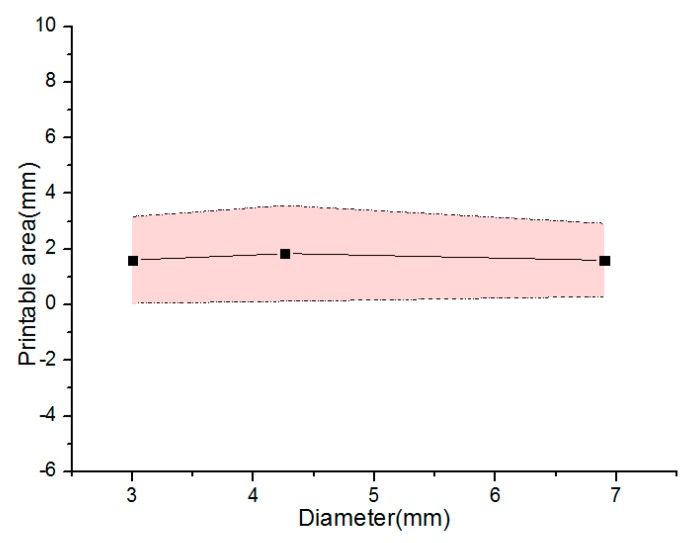
The relationship between the printable area and diameter under the different extrusion pressure.

Figure 7 shows the relationship between the printable area and extrusion pressure under the different diameters. We can see that the relatively stable thickness region can be derived to analyze the forming state of vessel-like structures. Based on this, the parameter of the theoretical model can be readjusted to estimate the thickness of vessel-like structures. Based on these analysis results, we can draw a conclusion that the optimized model parameter ∇ is derived

(5) H=∇β

where β is $\alpha \sqrt{{\left(V_{3}C/2V_{2}\right)}^{2}-L^{2}}-r$, ∇ is the optimized parameter ranged from 0.45 to 2.45.

**Figure 8:**
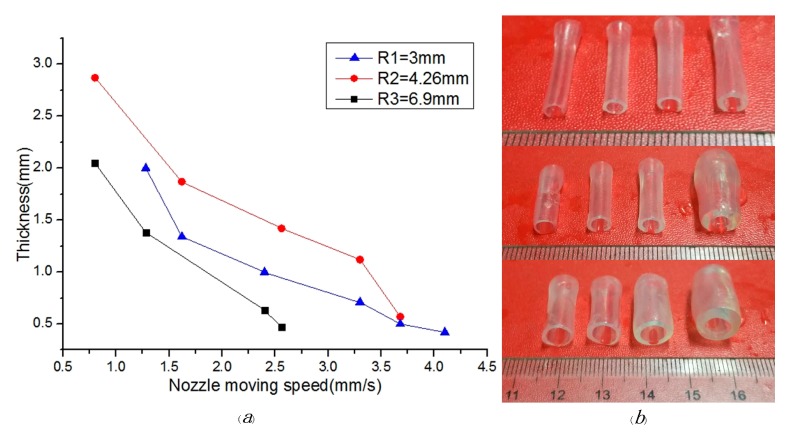
The molding relationship and effect: (**a**) dependence of the nozzle moving speed on thickness under the different diameters; (**b**) the relationship between molding effect and diameter.

Dependence of the moving speed of nozzle on molding effect: The controllability of materials plays a significant role in the construction of vessel-like structures. In the case of high pressure, the materials are difficult to be controlled in this 3D printing system. Meanwhile, the materials are difficult to be extruded under low pressure circumstances. To overcome the above-mentioned drawbacks, the pressure and the rotating speed were set to 0.224 MPa and 4.25 r/s, respectively. The vessel-like structures have the different thicknesses under the different nozzle moving speed. The relationship between thickness and moving speed of nozzle is shown in Figure 8. The conclusion is that the thickness is on the decline with the increase of nozzle moving speed. When the nozzle moving speed is less than 0.75 mm/s, the vessel-like structure will present material accumulation. Conversely, when the nozzle moving speed exceeds the certain values, vessel-like structures will not form.

**Figure 9:**
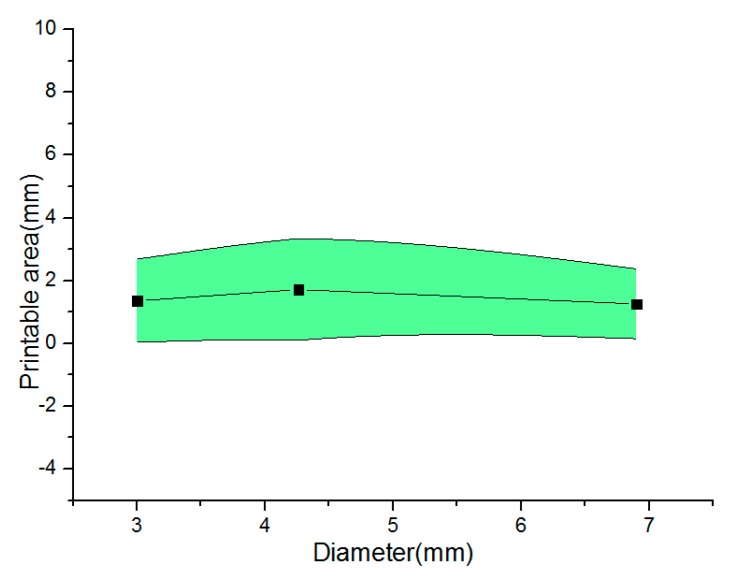
The relationship between the printable area and diameter under the nozzle moving speed.

Parameter optimization design: Figure 9 shows the relationship between the printable area and the different diameters under different nozzle moving speeds. Similarly to the Figure 7, the vessel-like structures have a relatively stable thickness region. Based on this, the parameters of the theoretical model can be readjusted to estimate the thickness of vessel-like structures.

(6) H=δμ

where μ is $\alpha \sqrt{{\left(V_{3}L/2V_{1}\right)}^{2}-L^{2}}-r$, δ is the optimized parameter ranged from 0.447 to 2.44.

Based on the optimized analysis results under the different pressure and nozzle moving speed, the optimized parameters of the theoretical model can be derived from the rotating forming device. The parameter of pressure is similar to the parameter of nozzle moving speed, namely δ≈∇. It is proven that the rotating rods with different diameters have a similar range under constant viscosity. Although the thickness characteristics of vessel-like structure are similar between the different pressures and nozzle moving speeds, we will intend to control the nozzle moving speed under a constant pressure due to the time delay in the process of material extrusion.

## 6. Conclusions

In conclusion, we have designed a novel 3D printing system using a rotary forming device to fabricate vessel-like structures for theoretical analysis, and its feasibility has been shown in this study. Compared with the deposition modeling method, those vessel-like structures produced by rotary forming method not only have higher forming accuracy, but also have faster molding speed. Using this rotary forming device, we have proposed a theoretical model to analyze motion synchronization. The experiments with alginate–gelatin solution are carried out to verify the theoretical results. The experiment results show that the thickness cannot be adequately predicted by the theoretical model and the thickness can be controlled by changing the correlation parameters. We have optimized the parameters under different pressures and nozzle moving speeds in the process of 3D printing, in which the parameter of pressure is similar to the parameter of nozzle moving speed, namely δ and ∇. This optimized theoretical model can be used to predict the thickness of the alginate–gelatin material. Meanwhile, it has been proven that extrusion-based 3D printing with alginate and gelatin could afford an opportunity to treat cardiovascular disease due to the possibility of angiogenesis in tissue engineering and organ regeneration.

## Figures and Tables

**Table 1:** The specification of 3D printing system.

Parameters	Value
Dimensions (*X* × *Y* × *Z*)	150 × 150 × 150 mm^3^
Position resolution	±5 μm
Temperature range	0–60 °C
Print speed	0.1–50 mm/s
Pressure range	0–1 MPa

**Table 2:** The parameters of experiment materials.

Concentration of Alginate (% *w*/*w*)	Concentration of Gelatin (% *w*/*w*)	Diameter (mm)	Calcium Chloride (% *w*/*w*)
3%	4%	3	5%
3%	6%	4.26	
3%	8%	6.9	
